# Clinical Utility of Advanced Microbiology Testing Tools

**DOI:** 10.1128/JCM.00495-19

**Published:** 2019-08-26

**Authors:** Melissa B. Miller, Faranak Atrzadeh, Carey-Ann D. Burnham, Stephen Cavalieri, James Dunn, Stephen Jones, Charles Mathews, Peggy McNult, John Meduri, Chris Newhouse, Duane Newton, Michael Oberholzer, John Osiecki, David Pedersen, Nicole Sweeney, Natalie Whitfield, Joe Campos

**Affiliations:** a University of North Carolina School of Medicine, Chapel Hill, North Carolina, USA; b Curetis USA Inc, San Diego, California, USA; c Washington School of Medicine, St. Louis, Missouri, USA; d Creighton University School of Medicine, Omaha, Nebraska, USA; e Texas Children’s Hospital, Houston, Texas, USA; f ClearView Healthcare Partners, Newton, Massachusetts, USA; g American Society for Microbiology, Washington, DC, USA; h Accelerate Diagnostics, Inc., Tucson, Arizona, USA; i Roche Diagnostics Corporation, Indianapolis, Indiana, USA; j University of Michigan Medical School, Ann Arbor, Michigan, USA; k Illumina, San Diego, California, USA; l Becton Dickinson, Diagnostics Systems, Sparks, Maryland, USA; m Genmark Diagnostics, Inc., Carlsbad, California, USA; n Children’s National Hospital, Washington, DC, USA; Emory University

**Keywords:** clinical utility, evidence, health economics, molecular methods, outcomes research, reimbursement

## Abstract

Advanced microbiology technologies are rapidly changing our ability to diagnose infections, improve patient care, and enhance clinical workflow. These tools are increasing the breadth, depth, and speed of diagnostic data generated per patient, and testing is being moved closer to the patient through rapid diagnostic technologies, including point-of-care (POC) technologies. While select stakeholders have an appreciation of the value/importance of improvements in the microbial diagnostic field, there remains a disconnect between clinicians and some payers and hospital administrators in terms of understanding the potential clinical utility of these novel technologies. Therefore, a key challenge for the clinical microbiology community is to clearly articulate the value proposition of these technologies to encourage payers to cover and hospitals to adopt advanced microbiology tests. Specific guidance on how to define and demonstrate clinical utility would be valuable. Addressing this challenge will require alignment on this topic, not just by microbiologists but also by primary care and emergency room (ER) physicians, infectious disease specialists, pharmacists, hospital administrators, and government entities with an interest in public health. In this article, we discuss how to best conduct clinical studies to demonstrate and communicate clinical utility to payers and to set reasonable expectations for what diagnostic manufacturers should be required to demonstrate to support reimbursement from commercial payers and utilization by hospital systems.

## Abstract (precis)

Advanced microbiology technologies are rapidly changing our ability to diagnose infections, improve patient care, and enhance clinical workflow. These tools are increasing the breadth, depth, and speed of diagnostic data generated per patient, and testing is being moved closer to the patient through rapid diagnostic technologies, including point-of-care (POC) technologies.

## INTRODUCTION

Until recently, clinical microbiology diagnostic techniques have relied almost exclusively on culture and isolation of microbes from patient specimens, with biochemical and phenotypic analysis to identify pathogen(s) causing infections (1–3). While creating the infrastructure for clinical laboratory scientists to perform culture and conduct analyses was costly, once well established, these techniques are inexpensive and can be highly standardized. Unfortunately, these techniques are time consuming, typically requiring 2 to 5 days or more to identify pathogens. In select cases, culture techniques may fail to adequately grow certain etiologic agents (e.g., Mycoplasma pneumoniae) or detect a viral pathogen. While developments in this space have historically focused on automating the culture process to reduce hands-on time and overall turnaround times to diagnosis, the turnaround time remains slow, which often causes physicians to treat empirically before diagnostic confirmation. Extended empirical treatment time may lead to the inappropriate use of antimicrobials, which may further contribute to the growing burden of antibiotic resistance. Studies have estimated that 30% to 50% of prescribed antimicrobials may be overprescribed or unnecessary (4, 5), which may contribute to the spread of infections due to increased antimicrobial resistance. Use of systemic antibiotics can lead to a disruption in the microbiome that can result in diarrhea and other complications (6, 7). One study concluded that some 20% of patients receiving antibiotics experienced an adverse drug event (8). The goal, therefore, is to avoid unnecessary antibiotics in addition to getting the patient on the most appropriate antibiotics when necessary.

In recent years, the introduction of new technologies has positively impacted both the diagnosis and treatment paradigms for infections. These tools are in the process of revolutionizing clinical microbiology testing in various settings. These include technologies such as matrix-assisted laser desorption–ionization time of flight mass spectrometry (MALDI-TOF MS), multiplex molecular diagnostic panels, and innovations that bring nucleic acid amplification testing to the point of care. The proteomic-based technology MALDI-TOF MS has seen wide adoption, particularly among academic hospitals. While this method still requires isolation and culture of pathogens, MALDI-TOF MS allows for the identification of a specific microbe based upon its unique proteomic fingerprint (9, 10). MALDI-TOF MS has led to significant time and cost savings, as correct diagnoses are made more rapidly without the need for additional confirmatory tests. Multiplex molecular diagnostic panels (11, 12) are also being introduced more commonly for a variety of conditions, including sepsis and nonspecific syndromes, such as respiratory or gastrointestinal (GI) infections. Multiplex assays can combine tests for numerous pathogens and resistance markers in a single panel, which can significantly reduce the time to diagnosis and, in select situations, bypass the need for culture. Furthermore, improvements in engineering and technology have also led to the development of improved point-of-care (POC) tests (13–16), which are poised to significantly impact the future treatment paradigm for many infectious conditions, such as viral respiratory infections and sexually transmitted infections (STIs). Low-complexity POC tests allow for nonlaboratory personnel (e.g., nurses and physician assistants) to conduct tests at the initial site of care, potentially allowing physicians to administer the therapy at the initial consultation and eliminating the need for follow-up assessments.

These tools have the potential to address many key challenges in the field of infection management by reducing the time to diagnosis and informing earlier therapeutic decisions, which may improve clinical decision making, patient outcomes, workflow, and antimicrobial stewardship (5, 17–19). These types of innovations also have the potential to significantly improve both individual patient outcomes and broader public health by facilitating better tracking of pathogens and changes in/development of antibiotic resistance (2).

However, in many cases, the clinical deployment of these technologies has been restrained by skepticism from payers and hospital administrators over clinical, and ultimately cost, benefits. Select referenced studies are underpowered from a statistical perspective, which may not demonstrate a benefit as clearly as would be desired. In some cases, advanced microbiology tests provide limited improvement in accuracy over standardized laboratory culture-based tests, although they provide workflow and efficiency benefits. Therefore, it is imperative to demonstrate robust evidence of clinical utility in a timely and cost-effective manner to increase our understanding of the benefits of adoption of advanced microbiology tests across care settings. More robust studies of clinical utility will also improve our knowledge of the impact on clinical outcomes and operations, which can lead to enhancements in care.

## OVERVIEW OF CLINICAL UTILITY AND EVIDENCE DEVELOPMENT CONSIDERATIONS

Clinical utility of a test is related to the added value it has for patient management. A test has utility if its results (positive or negative) provide information that is of value to the patient and the provider in making decisions about effective clinical care. It can take the form of improved efficiency in clinical decision making, streamlined clinical workflow, better patient outcomes, and/or cost offsets or avoidance (20–24). The level of clinical utility evidence required will likely depend upon a variety of factors, including the current standard of care (SOC), the setting of care, and potential cost offsets to mitigate the added cost of care, as well as the magnitude of the cost of the test itself.

Clinical utility, in the microbial space, is considered to include instances in which new approaches can inform treatment decisions by providing information to key stakeholders, such as the patient, physician, and payers, to diagnose, monitor, and/or predict disease progression. For example, rapid POC influenza testing has been shown in several studies to significantly reduce prescriptions of antimicrobials and increase prescriptions of oseltamivir (Tamiflu) in outpatient care clinics (25–27). Rapid POC influenza testing can improve antibiotic stewardship and positively impacts patient management via a faster resolution of flu symptoms. Better initial treatment decision making could also influence important outcomes, like morbidity and mortality, for at-risk patient populations, such as the elderly and immunocompromised.

It is important to note that a necessary first step for any new diagnostic is to demonstrate that it meets, and potentially surpasses, the bar for accuracy of the current SOC. However, accuracy alone does not, in and of itself, demonstrate clinical utility. Instead, it is a prerequisite for utilization that facilitates impact on clinical care, which can then translate into clinical utility.

It is important that microbiologists conducting clinical utility studies consider which type of trial is most appropriate for the technology and endpoint they wish to study, as well as the ultimate stakeholder audience for the diagnostic technology. Whenever possible, studies designed to generate evidence of clinical utility should consider the needs of potential patient populations. For instance, hospital administrators often look for evidence generated in a system that closely resembles their organization, to provide confidence that the clinical utility demonstrated in a trial may translate to real-world experience. Also, many hospital systems may conduct their own trials with new diagnostic technology, to provide real-world evidence for improved workflow, decision making, and patient care resulting from the adoption of new technology. Published results are impactful for commercial payers when they can demonstrate clear changes in clinical decision making for patients that are representative of their plan members that were directly facilitated by the information provided by the test. Ideally, payers would like to see data demonstrating that these decisions correlated with positive clinical outcomes. However, payers are aware that many factors go into clinical outcomes beyond the diagnostic result, and therefore it may be sufficient to simply show a connection between the changes in decision making and potential clinical outcome.

Once the audience is defined, the study designers also need to determine the appropriate level of evidence. The key considerations are the overall size of the trial and its representativeness. The size of the trial relates to the relative rarity of the events that occur. Statistical power calculations can be done to determine the number of patients needed relative to the delta in a change of endpoint. As the numbers of groups of patients and parameters to be measured increase, the number of patients needed to achieve a significant result will also increase. For instance, a study that measures only one patient group (e.g., high risk for respiratory conditions) with two diagnostic arms (e.g., SOC compared to multiplex panels) may be able to reach significance with 100 patients. A trial that was following three cohorts of patients (e.g., high risk, low risk, and the general population) and measuring three diagnostic arms (e.g., culture and phenotype, single-gene PCR tests, and multiplex molecular panels) would likely require 800 or more patients (28, 29). A study of this complexity and size may not be feasible for many clinical labs.

To illustrate representativeness, it is important to conduct the trial at a location which is comparable to the broader clinical community. For instance, by testing MALDI-TOF MS technology in large academic hospitals with a significant volume of testing, the investigators were able to demonstrate the significant clinical utility of MALDI-TOF MS to improve workflow and decision making in central laboratories of major hospital systems (30, 31). When researchers conduct studies in hospital systems with a unique patient population or practices, it may be challenged by payers and others as being nonrepresentative.

## OVERVIEW OF CLINICAL UTILITY EVIDENCE DEVELOPMENT CONCEPTS

**FIG 1:**
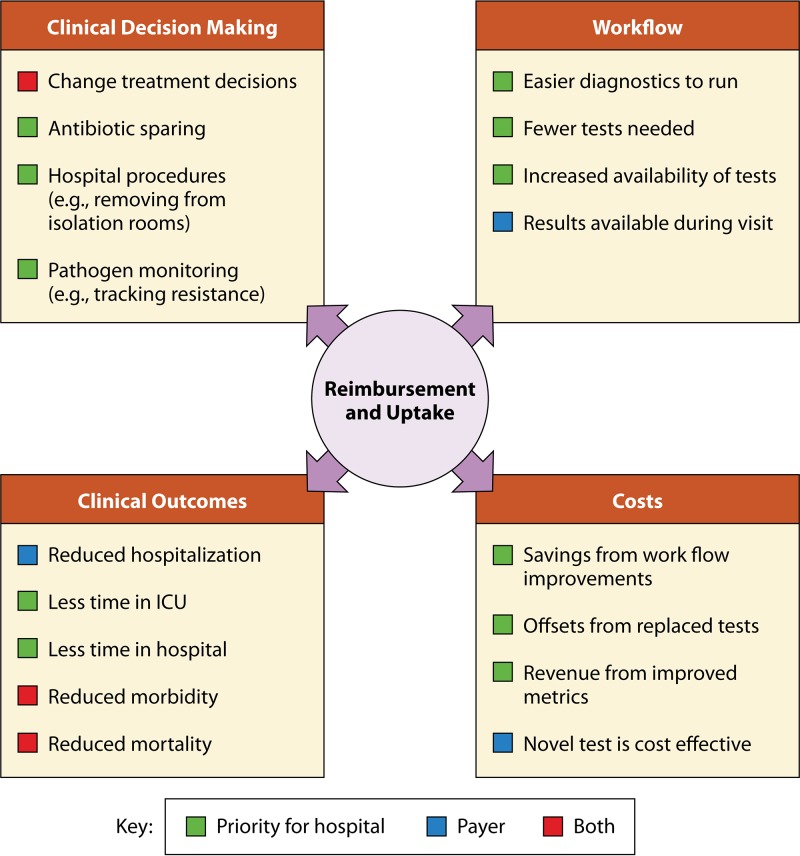
Endpoints for studies of clinical utility.

While overall clinical utility for microbial diagnostics covers the areas defined above, the value proposition of technology may typically be demonstrated by impacting one or more of the following categories: clinical decision making, clinical workflow, patient outcomes, and clinical costs (Fig. 1).

The first step is defining the current standard of care; researchers should always strive to test clinical utility in comparison to the current SOC, with the goal of significantly improving at least one step of the diagnostic paradigm. Therefore, microbiologists should carefully study the SOC and determine where the new test can make the most significant improvement (32). If current care is well defined, an observational trial measuring changes in care could be sufficient. If the current SOC is not well defined, these types of studies will be most effective if they are large prospective studies over multiple institutions, allowing for the determination of the clinical utility of such a test in a variety of settings with different care paradigms.

**FIG 2:**
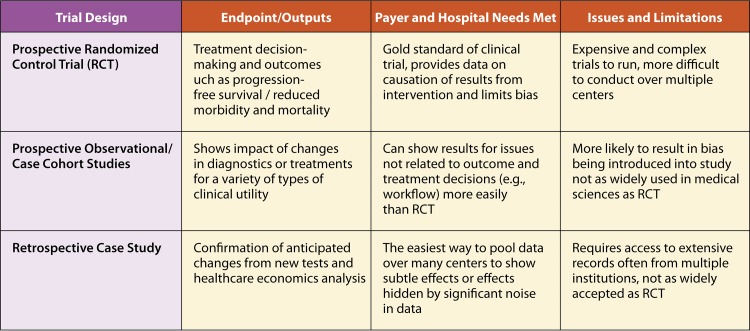
Select examples of types of trials that can be used to demonstrate clinical utility.

Researchers can demonstrate clinical utility through a variety of trial designs (33), including randomized control trials (25, 34), in which patients are randomly assigned as they enter the health care system to a new diagnostic being tested or to the SOC (Fig. 2). At the end of the study period, the researchers can ascertain how measured endpoints varied between the two groups. Prospective interventional or observational trials (17, 19, 35, 36) are conducted by measuring a specific endpoint(s) for a selected length of time before introducing the new diagnostic. After the new diagnostic has been established, the same endpoint(s) will be measured over a similar period so that changes caused by the diagnostic can be ascertained. Retrospective analysis trials (37–39) are conducted once a test has been widely deployed for an extended period of time; researchers can then gather historical data for the endpoint(s) either prior to the deployment of the test or from locations where it has not been deployed and compare these endpoint data to data from similar institutions where the test is in place. Finally, one can collect information about the impact of test on clinical decision making using a pretest/posttest survey instrument. In this type of study, a physician is asked to record their current care plan based on available information. They are then presented with the test results and asked if their decision would change. Pretest/posttest survey instruments are helpful in that they capture the actual shifts in thinking that occurs as a result of the test, but it is limited in that it does not track actual behavior or outcomes moving forward.

Although a particular trial design may be more appropriate for a select technology or setting of care, all of these types of trials can be appropriate ways to demonstrate clinical utility. Historically, randomized control trials have been considered the gold standard for evidence generation and still are in many circumstances, especially for pharmaceutical agents. Select commercial payers, likely informed by their experience with pharmaceuticals, may expect randomized control trials for advanced microbial diagnostics, although there is a growing understanding that each of the trial types has advantages in certain situations but may not be required or may have limitations in others. For example, it is difficult to execute a diagnostic trial with a “placebo” test. In particular, diagnostic tests can show their value through retrospective and prospective observational studies, in which researchers compare the care delivered by a novel technology to a historical standard of care.

### Clinical decision making.

Improvements in clinical decision making are often the primary endpoint of diagnostic studies, due to both feasibility and the fundamental purpose of diagnostics, which is to inform better the treatment choices physicians make. It is important to note that improved clinical decision making can create value by not just improving therapy decisions and supporting antibiotic stewardship efforts but also through conserving resources (e.g., faster emergency room [ER] triage, or removing patients who do not have communicable infections from isolation rooms).

When studying changes in clinical decision making resulting from the implementation of new technologies, common endpoints that may be examined include measuring time to initiation of appropriate therapy, time to initiation of antibiotic escalation/de-escalation protocols, degree of expedited ER triage for hospital admission or discharge, the time patients spend in isolation rooms, and effective antibiotic stewardship metrics. For instance, several successful studies have been completed using MALDI-TOF MS in central hospital laboratories, demonstrating changes in types of antimicrobials prescribed and reduced time to placing patients on appropriate antibiotic regimens (19, 35, 40, 41).

Technologies that impact the removal of patients from isolation rooms or critical treatment decisions (e.g., for patients with sepsis) need to show statistical impact for instances and causation for important decisions (4, 17, 19). For example, a recent study of improved decision making from new diagnostic technology in the management of sepsis showed via a randomized controlled trial that multiplex panels have hastened the diagnostic process and led to the faster placement of patients on appropriate antimicrobials (40). These changes in decision making result in, at a minimum, improvement in antibiotic stewardship, and they may over a longer time frame reduce mortalities related to sepsis (34, 42, 43).

As technologies become more widely used or are being applied to more common decision points, retrospective and prospective studies become more feasible and are easier to execute. For example, the greatest utility of POC influenza tests for changing clinical decision making is evident with studies in ERs, primary care physician offices, and outpatient clinics, by showing reductions in empirical antibiotic treatment and increases in Tamiflu prescriptions in these clinical settings through larger retrospective and prospective studies (25, 44, 45). Similarly, a study of a gastrointestinal panel showed reduction of several patient days on antibiotic and in the length of time to discharge (46). In the future, multiplex syndromic panels for outpatients may develop evidence of clinical utility by targeting specific high-risk groups, such as immunocompromised and pediatric patients and elderly patients in assisted living facilities. Hospital admission is a critical decision point for these patients and therefore a potential endpoint; expediting hospitalization of patients with severe infections may significantly improve health outcomes. Furthermore, some patients with less severe infections may benefit from avoiding hospital admission, limiting the potential of nosocomial infections and avoiding the sizable cost of hospitalization. Information about the use of these technologies can be collected through collaborative efforts in multiple institutions or from a single institution that works with high volumes of vulnerable patients.

### Clinical workflow.

Clinical workflow is substantially different in the outpatient and inpatient settings and typically is of higher priority for inpatient services. For hospitals, enhancing clinical workflow has the potential to significantly improve patient care and lead to lower costs, particularly as more complex and/or labor-intensive tests may be replaced with simpler and/or more efficient tests. Also, such improvements can aid hospitals in meeting important quality metrics, such as limiting hospital-acquired infections and improving antimicrobial stewardship. Improvements in the workflow can be accomplished by streamlining the diagnostic process or by more rapidly monitoring changes in antimicrobial resistance or types of pathogens present (19). Many hospital central laboratories are limited to running tests for select pathogens at certain times of the day and/or week, due to the complexity of tests which require highly trained technologists to perform the assays. Also, there may be periods of higher volumes (e.g., influenza season, disease outbreak, etc.) where central laboratories can be overwhelmed by the workload. High-volume laboratory demands can have further downstream impacts, including delaying the ability for other diagnostic tests and clinical services in the hospital to be efficiently run. New advanced microbial testing tools offer the potential to significantly improve workflow in both outpatient clinics and laboratories by decreasing the time and technical expertise required to perform each test. Also, advanced technologies may allow an increased number of patient samples to be tested simultaneously, thereby increasing efficiency and throughput of the laboratory, resulting in improved workflow and faster results. Unfortunately, clinical workflow is rarely quantified and reported in the literature, making comparisons across institutions difficult. More studies in this area would significantly benefit the field. Relevant endpoints tested for clinical workflow could include time to reaching a confirmed diagnosis, number of tests that must be run for diagnosis, clinical laboratory hours worked per diagnosis, number of samples the laboratory processes per unit time, and the frequency at which backlogs develop in the laboratory. Furthermore, it is important to note that the United States is currently facing a shortage of trained clinical laboratory personnel that is not expected to improve in the immediate future. Technologies that allow fewer technicians to run more tests will likely play an essential role in overcoming this challenge (47–49).

One of the most significant ways for improvements in outpatient workflow clinical utility is to show that clinics can triage patients faster and thereby increase the volume of patients assessed and treated in a similar time frame. Increasing the volume of patients a physician can see in a select amount of time has the potential to improve patient access by allowing a physician to see more patients per day (50, 51). For example, when a POC test identifies a patient requiring immediate treatment while also identifying individuals that are safe to release, it could significantly improve workflow by diagnosing and treating patients in a single visit without the need for follow-up assessments if the diagnosis is delayed (e.g., sexually transmitted infection [STI] tests, HIV, influenza, group A *Streptococcus*, etc.). POC tests can not only identify patients with severe infections that need to be admitted to the hospital but can also identify patients with more minor conditions that are candidates for discharge and therefore reduce occupation of ER beds (50, 51). One key consideration is that often these decisions are made quickly, so to impact clinical workflow, the results must be made available within a limited time frame (e.g., during or immediately after the visit). Prospective or retrospective studies may be sufficient to demonstrate altered patient flow due to the utilization of a new diagnostic technology. Appropriate endpoints for outpatient workflow could include time to diagnosis for outpatients, number of patients seen, number of tests/diagnostic procedures performed, duration of visit per patient, and frequency for which follow up visits are needed. An example of this type of study can be found in a recent publication describing a randomized control trial in which the benefits of same-day testing for chlamydia and gonorrhea were evaluated compared to SOC testing (2- to 3-day turnaround time). In this study, there was 0% undertreatment compared with 43.8% for patients tested by the SOC. (52) A retrospective study of a multiplex molecular gastrointestinal (GI) testing panel showed not only a reduction in antibiotic prescription but also fewer endoscopic and abdominal radiology procedures (46).

### Patient outcomes.

Patient outcomes are a central focus of medical studies and are typically focused on reductions in morbidity and mortality. Microbial diagnostics may meaningfully improve patient outcomes, as a more rapid diagnosis will likely directly impact timely clinical decision making and improve overall patient care (19, 36, 53, 54). While these benefits may represent evidence for clinical utility, it is often difficult to demonstrate that improved outcomes are specifically due to the diagnostic test, given the multitude of factors associated with patient therapeutic response. The diagnostic tool itself should only be held to the standard of informing the correct treatment decision and should not be required to prove that the therapeutic positively impacts care, as limitations in the treatment can overwhelm improvements in diagnostics and many benefits of decision making (e.g., antibiotic sparing) will not result in morbidity and mortality improvements. It should be a given that when a pathogen is correctly identified and the optimal treatment is initiated that an improvement has occurred, whether or not this translates into a measured direct reduction in morbidity and mortality. Therefore, the most appropriate approach may be for diagnostic developers to demonstrate an improvement in clinical decision making for reimbursement.

These efforts can initially be focused on the demonstration of clinical utility in smaller patient populations that are more likely to benefit from diagnostic improvements, such as infants, immunosuppressed or compromised patients, and the elderly. For instance, multiplex molecular syndromic panels might show significant improvements for immunocompromised patients by quickly identifying those that should be treated immediately for serious infections.

Clinical outcomes are of paramount importance to both inpatient and outpatient care. However, the particular outcomes desired can be different in each setting. For inpatient care, the most common desired outcomes will be reducing the length of hospital stay, the time patients spend in the intensive care unit (ICU), readmissions, and, ultimately, infection-associated mortality. For example, MALDI-TOF MS, when conducted post positive blood culture has shown a reduction in mortality for patients with sepsis in prospective interventional trials (19). However, in the inpatient setting, patient outcomes may be dependent on multiple factors other than diagnostics, particularly for more serious infections or for patients with complex comorbidities. Studies of patient outcomes, therefore, are often assessed with prospective or retrospective clinical trials, as they will need to be conducted over several institutions or conducted for long periods of time to generate sufficient statistical power to reach conclusions on a diagnostic-specific impact on patient outcomes.

For outpatient care, critical endpoints regarding patient clinical outcome include situations in which the identification and management of infections avoid hospitalization and development of more serious conditions. In many circumstances, changes in clinical decision making that would reasonably be expected to improve outcomes along with hospital admission rates will be appropriate endpoints. However, once a diagnostic is widely used in the outpatient setting, large retrospective studies over many medical systems using the technology will likely be feasible.

### Cost-benefit evaluation.

Underlying each of these categories is a fundamental question about whether the additional clinical benefits can be justified from a financial perspective. In the United States, this takes the form of cost/impact and simple return on investment analysis rather than formal cost-effectiveness evaluations as seen in other markets. Advanced technologies usually come at some additional cost, but they have the potential to free up resources by reducing the use of other tests, or by avoiding additional diagnostic procedures. It is worth remembering that different stakeholders in the hospital may be motivated by different cost-benefit considerations; for instance, laboratory directors may be interested in how cost translates to efficiency, while C-suite executives will likely focus more on return on investment.

In the inpatient setting, cost will be less of a concern because inpatient care is focused on more serious infections with severe/costly outcomes. Also, there are more ways for costs to be offset in inpatient care. For instance, studies with multiplex gastrointestinal panels have shown some ability to reduce cost by removing patients from high-cost isolation rooms and moving them to general wards when the patients were shown not to have communicable infections (5). Other studies have shown cost reductions through fewer diagnostic testing/imaging studies and reduced length of stay, not only in GI patients but in those with the respiratory virus as well (46, 55). Another example is the use of MALDI-TOF MS, which has routinely demonstrated lower costs per sample than culture and phenotype assays for organism identification. However, the high upfront cost of MALDI-TOF MS instruments means these savings might only be realized by high-volume laboratories. Appropriate endpoints for measuring cost are direct spending changes in dollars, but more sophisticated health economic modeling showing differences in quality-adjusted life years/incremental cost-effectiveness ratios may be justified (4). It is worth noting that cost avoidance is often harder to quantify and track than direct costs and revenue, which makes cost savings benefits from a test more difficult to communicate to payers and users.

For outpatient care, direct costs are unlikely to show clinical utility, as tests will be compared to relatively inexpensive methods with limited ability to create offsets. In this situation, cost becomes a barrier, leading commercial payers to be less enthusiastic to reimburse a test because of the negative economic impact it may have. Of particular note, high-priced tests are more likely to be held to higher evidentiary thresholds by commercial payers, who may demand larger clinical trials or randomized control trials, when at a lower price point prospective observational or retrospective trials would have been accepted.

## DISCUSSION OF WAYS TO FOSTER CLINICAL UTILITY EVIDENCE DEVELOPMENT AND INNOVATION

### Role of evidence.

To effectively maximize clinical uptake and broad payer coverage of advanced technologies, the microbiology community must collectively form an action plan involving a variety of stakeholders to collaborate and demonstrate the beneficial effects of advanced technologies in the management of infectious diseases. A key starting point for collaborative efforts in the microbial diagnostic space would be to conduct studies to determine the economic and clinical challenges and limitations of the current diagnostic paradigm. Properly conducted studies on this topic could help persuade payers of the potential room for growth in this area and will set the stage for the possible benefits of new technologies that can result in improvements in patient care.

Key stakeholders should work closely with publishers of clinical guidelines to articulate the role and best practices for the use of these tools to better inform payers on the value and practicality of new tests. The continued generation of appropriate clinical data may also lead to a willingness to include advanced technologies in published guidelines. Including new diagnostic technologies in clinical guidelines would add significant value, particularly in the eyes of payers, who refer to published guidelines to inform their decision on whether to reimburse a technology. Clear guidelines are particularly important for those technologies where the added cost of a technology is currently perceived as a limitation for wide adoption in routine practice (e.g., next-generation sequencing [NGS] technologies for microbiology).

Prioritizing clinical utility evidence generation by hands-on users in real-world settings, such as improved impact on everyday clinical decision making and individual patient outcomes, will be important to drive the future value proposition of advanced microbiology technologies. While this additional evidence will likely play a major role in facilitating broader utilization of new diagnostics among health care entities, it will also educate payers on the added value to encourage broader payer coverage and reimbursement.

It may be possible to eventually demonstrate outcomes not only at the individual patient level but also at the population level. For example, there is potential to show that outcomes are improved via public health benefits that result from better antibiotic selection and community-acquired resistance management through the regional applied use of advanced diagnostic technologies. The full benefits of these technologies will only be realized once antimicrobial stewardship and operational improvements (e.g., strain tracking, hospital infection control surveillance, etc.) are applied in aggregate. This will require a greater collaborative/coordinated effort across multiple institutions potentially coordinated by a public health entity. These types of studies will require collaboration between many stakeholder organizations, such as the American Society of Microbiology (ASM), clinical and physician societies, and government groups. When these types of studies are conducted, positive results should be utilized to encourage the Centers for Disease Control and Prevention (CDC) to publish formal guideline updates encouraging widespread adoption of a diagnostic technology, given the significant improvements in patient outcomes and potentially in public health.

### Role of the microbiology and infectious disease community.

As noted above, many of the forms of clinical utility require showing the impact on clinical decision making; therefore, it is important for microbiologists to understand how clinical decision making is done in the current SOC by engaging clinicians managing infections. Success in developing clinical utility information will require microbiologists building bridges to members of the clinical community. The clinical stakeholders needed include not only infectious disease specialists but also infection control practitioners and primary care and emergency room physicians to determine the impact of these tools on everyday care. Better coordinated action requires findings consensus about the key benefits and required evidence and jointly and clearly articulating this information to key stakeholders, such as hospital administration and payers.

### A role for industry.

Both the pharmaceutical and diagnostic manufacturing industries will also be required to be involved in orchestrating the generation of clinical utility evidence. Given that hospital administrators often prefer to undertake a trial period with new technologies to gain first-hand experience, diagnostic technology manufacturers may need to pursue collaborations for this to be actively achieved. Furthermore, they can work to guide not only microbiologists but also facilitate early partnership with those in clinical and financial roles about the design of studies which could help illustrate the clinical utility of these deployed tools in a fair and balanced way. They can also play a role in helping community hospitals understand where to find clinical utility information and how to share clinical utility information so that advanced care approaches are not limited to academic medical centers. This support can come in the form of research grants that are specifically for utilization reviews rather than for traditional clinical trials. Critically, the microbial diagnostic industry should recognize that efficacy and clinical utility trials conducted by industry are often viewed skeptically by clinicians and payers. A better solution may be to provide funding to institutions using the technology to support studies of clinical utility and best practices that the institutions can publish independently.

Additionally, pharmaceutical leaders in the microbiology space may be required to actively participate in data gathering and publication supporting the concept that next-generation antimicrobials may be more effective, particularly if paired with the most advanced diagnostic technologies. This will likely require active collaboration between pharmaceutical and diagnostic companies to ensure the clinical utility benefit of appropriate prescribing of next-generation treatments is influenced by novel technologies entering the space. The National Institutes of Health (NIH) and the CDC have the potential to facilitate and mediate these types of collaborations through improving communications, providing funding for important studies, promoting a strong clinical research environment, and supporting inclusion in clinical guidelines when tests are shown to be effective and economical.

### A role for government and advocacy.

Demonstrating the value of novel microbiology technologies will likely require a holistic approach to be undertaken by the microbiology community. Engaging key agencies, such as the CDC and NIH, to increase funding for large studies to generate large data sets of evidence will be a key strategy to articulate the message. Such studies involving a variety of stakeholders should aim to demonstrate improved antibiotic stewardship, patient outcomes, and communicate the overall economic and health benefits for the community following the adoption of novel microbiology technology in the future. For example, the CDC has responded to the U.S. National Action Plan for Combating Antibiotic-Resistant Bacteria by launching initiatives such as Antibiotic Resistance (AR) Solutions, which involves investments in national infrastructure to prevent resistant infections (https://www.cdc.gov/drugresistance/solutions-initiative/index.html). We urge the CDC to include evaluation of advanced diagnostic technologies as potential tools to improve antimicrobial stewardship, clinical decision-making/workflow, clinical outcomes, and the detection, tracking, and prevention of resistant infections.

Professional societies, such as ASM, will likely play a key role in developing close working relationships with government organizations, such as the NIH and CDC, to emphasize the value of clinical utility of advanced microbial diagnostics. Importantly, these groups should focus on supporting the development of evidence and sharing of information in areas that are not a high priority to any single stakeholder to help resolve collective action issues. Moreover, demonstrating robust clinical utility will likely require clinical microbiologists to engage with each stakeholder type, from a variety of physician groups to hospital administrators and payers, to aid understanding and communicate the potential benefit that advanced microbiology tools provide in various care settings. These improvements may be achieved by improving not only the speed and accuracy of disease diagnosis but also key characteristics such as workflow and cost avoidance (32).

### Implications for future incorporations of technology.

The advances and innovations in microbial diagnostic technologies over the last decade are beginning to have a significant impact on the way we diagnose and manage infectious diseases. In the coming years, an additional cohort of new microbial diagnostics is expected to enter the space. Technologies that include advanced genomics (56, 57), proteomics, and rapid susceptibility tests (58) are expected to cause dramatic changes by tackling some of the most important problems for microbial diagnostics. Additionally, advanced analytic tools, such as artificial intelligence and machine learning, can enhance the information extracted from the data these technologies collect (49, 59, 60).

For example, the menu of culture-independent nucleic acid amplification tests and syndromic panels is expanding. These advances will likely favor the deployment of culture-independent reporting of antimicrobial resistance (AMR) determinants, including the creation of a clear correlation of AMR genotype to antimicrobial susceptibility phenotype/MICs. Also, automated microscopy is being leveraged for early detection of sepsis by detection of morphological changes in monocytes indicative of dysregulated immune response or morphological changes in bacteria indicative of drug susceptibility (61).

Next-generation sequencing methods and proteomics (e.g., MALDI-TOF) are expected to impact key diagnostic segments in the future. In contrast to PCR panels, these methods have the potential for “hypothesis-free” detection of pathogens and host response markers. NGS-based analysis of pathogens further allows phenotypic prediction, such as detection of AMR determinants, virulence factors, and mobile genetic elements. Also, whole-genome sequencing of isolates by next-generation sequencing allows strain typing at nucleotide-level resolution for epidemiological studies and infection control. These methods have tremendous potential in the clinical microbiology lab, opening a novel paradigm for diagnostics.

However, to be deployed clinically and realize this potential, these technologies will need to build on efforts associated with more established technology that has demonstrated clinical utility. Our hope is that the concepts outlined throughout this paper will facilitate the demonstration of the clinical utility of recently launched novel methods so that the even newer tools and techniques, as described above, will be able to find a pathway to success and routine application in the clinical microbiology laboratory. Adoption of these technologies may also require hospitals and payers to place a higher priority on infection control than they do currently and to support their infection control centers.

## CONCLUSION

The need for improvements in microbial diagnostics and thereby in management of infectious disease is clear and urgent. This need has the potential to be filled by a combination of new technologies that have entered the diagnostic space or will enter it shortly. However, there is a clear gap in the field that is preventing these technologies from being widely deployed to fill the current unmet clinical need for rapid and improved testing. While the necessity of deploying better microbial diagnostics is not lost on microbiologists and infectious disease specialists, other key stakeholders have lower awareness. Therefore, a collective effort is needed from microbiologists and clinicians handling infectious diseases to communicate to other stakeholders the costs and downsides of the current SOC. Demonstrating and communicating how the low cost of phenotypic methods is often offset by the high cost of preventable morbidity and mortality that comes from a slow diagnostic SOC, and how new tests can directly impact and improve clinical decision making, is needed. Clearly defining and describing these issues to commercial payers, hospital administrators, and government regulators will smooth the deployment of these technologies and benefit individual and communal health.
